# Mitochondrial Complex 1 Activity Measured by Spectrophotometry Is Reduced across All Brain Regions in Ageing and More Specifically in Neurodegeneration

**DOI:** 10.1371/journal.pone.0157405

**Published:** 2016-06-22

**Authors:** Amelia Kate Pollard, Emma Louise Craig, Lisa Chakrabarti

**Affiliations:** School of Veterinary Medicine and Science, University of Nottingham, Sutton Bonington, United Kingdom; National Institute of Health, UNITED STATES

## Abstract

Mitochondrial function, in particular complex 1 of the electron transport chain (ETC), has been shown to decrease during normal ageing and in neurodegenerative disease. However, there is some debate concerning which area of the brain has the greatest complex 1 activity. It is important to identify the pattern of activity in order to be able to gauge the effect of age or disease related changes. We determined complex 1 activity spectrophotometrically in the cortex, brainstem and cerebellum of middle aged mice (70–71 weeks), a cerebellar ataxic neurodegeneration model (*pcd*^*5J*^) and young wild type controls. We share our updated protocol on the measurements of complex1 activity and find that mitochondrial fractions isolated from frozen tissues can be measured for robust activity. We show that complex 1 activity is clearly highest in the cortex when compared with brainstem and cerebellum (p<0.003). Cerebellum and brainstem mitochondria exhibit similar levels of complex 1 activity in wild type brains. In the aged brain we see similar levels of complex 1 activity in all three-brain regions. The specific activity of complex 1 measured in the aged cortex is significantly decreased when compared with controls (p<0.0001). Both the cerebellum and brainstem mitochondria also show significantly reduced activity with ageing (p<0.05). The mouse model of ataxia predictably has a lower complex 1 activity in the cerebellum, and although reductions are measured in the cortex and brain stem, the remaining activity is higher than in the aged brains. We present clear evidence that complex 1 activity decreases across the brain with age and much more specifically in the cerebellum of the *pcd*^*5j*^ mouse. Mitochondrial impairment can be a region specific phenomenon in disease, but in ageing appears to affect the entire brain, abolishing the pattern of higher activity in cortical regions.

## Introduction

Complex 1 is the largest of the five enzyme supercomplexes in the mitochondrial electron transport chain. Though it performs the major first step of the oxidative phosphorylation pathway it is still not completely understood partly due to the number of its sub-units and their potential interactions[1]. Interruption of the activity of complex 1 either by toxins such as rotenone, drugs like 1-methyl-4-phenyl-1,2,3,6-tetrahydropyridine (MPTP) or due to genetic disorders such as Leigh’s Syndrome or Leber hereditary optic neuropathy has debilitating consequences[2][3].

Many studies support the mitochondrial theory of ageing, in particular the hypothesised decreased functionality of the ETC: complex 1 is often cited as the most likely site of an ETC impairment [4] [5], [6], [7], [8]. Complex 1 is thought to be a site of impairment due to more of the subunits being encoded by mitochondrial rather than nuclear DNA. Mitochondrial DNA due to its proximity to reactive oxygen species produced by this organelle is proposed to be more susceptible to oxidative damage[9] [7], [10]. Complex 1 activity has been shown to decrease with age in various tissues when experimentally determined, most notably in rat brain and heart, where a strong positive correlation was demonstrated between a decreased complex 1 functionality and an increase in ROS production [8].

Decreases in complex 1 have also been demonstrated in diseases more frequently encountered in older age such as neurodegeneration; in particular Parkinson’s disease (PD) [11], [12]. Complex 1 began to be implicated in the aetiology of certain neurodegenerative disorders following an accidental intake of a complex 1 inhibitor– 1-methyl-4-phenyl-1,2,3,6-tetrahydropyridine (MPTP) by a group of drug addicts, who developed with symptoms unable to be differentiated from true Parkinson’s disease [13]. Complex 1 inhibitors such as rotenone produce similar pathology in rats providing excellent models to study neurodegenerative processes [12].

Though we already know that complex 1 decreases in the brain during ageing and neurodegeneration we set out to measure directly which gross anatomical region displays the greatest activity or undergoes greatest changes in aged or disease brains. Previous studies of rat brain suggest the greatest complex 1 activity exists in the cortex and cerebellum but others suggest that no differences are displayed, or that the cerebellum in fact has lowest measured activity [6], [14], [15].

The aim of this study was to establish and compare the levels of complex 1 activity in the three major brain compartments. Our study differentiates between the effects of ageing and of neurodegeneration by studying a mouse model that undergoes the neurodegenerative process at a young age[16].

To investigate complex 1 activity changes in neurodegeneration a mouse model was used; the Purkinje cell degeneration mouse (*pcd*^*5J*^). The *pcd*^*5J*^ mouse is a neurological autosomal recessive phenotype. Within 3 weeks of birth *pcd*^*5J*^ mice begin to lose their cerebellar Purkinje cells, by 4 weeks of age the animals have developed pronounced gait ataxia [17], [18], [19]. The *pcd*^*5J*^ mouse model is caused by a mutation in the Nna1/CCP1 gene [20]. Nna1 encoded proteins have been shown to be localised in mitochondria, and a loss of these proteins or their function results in mitochondrial dysfunction, including complex I activity deficits [21].

Despite an increased interest in mitochondrial function in ageing and neurological disease we found that the overall levels of complex 1 activity are not known in the three major compartments of the mammalian brain. In disease this information can be highly relevant since early in the process it may be that circuits in a non-diseased region of the brain can compensate for neurodegeneration. In normal ageing we wanted to know whether mitochondrial dysfunction measured via complex 1 activity affects the entire brain or one compartment more specifically. We show here that there are indeed significant differences in complex 1 activity between the brain compartments measured. The activity values change significantly with age and in neurodegeneration.

## Materials and Methods

Animals were bred and housed in accordance with strict Home Office stipulated conditions. The overall programme of work (in respect to the original UK Home Office Project Licence application) is reviewed by the Animal Welfare and Ethical Review Body at the University of Nottingham and then scrutinised by the UK Home Office Inspectorate before approval by the Secretary of State. Individual study protocols link to the overarching Home Office Project Licence and are made available to the Named Animal Care and Welfare Officer, the Named Veterinary Surgeon (both are members of the AWERB), the animal care staff and the research group. The Project Licence Number for the breeding and maintenance of this genetically altered line of mice is PPL 40/3576. The mice are typically group housed and maintained within solid floor cages containing bedding and nesting material with additional environmental enrichment including chew blocks and hiding tubes. Cages are Individually Ventilated Cage Units within a barrier SPF unit to maintain bio-security. Animals are checked daily by a competent and trained animal technician. Any animal giving cause for concern such as subdued behaviour, staring coat, loss of weight or loss of condition will be humanely killed using a Home Office approved Schedule 1 method of killing.

### Mouse tissues

Animals were divided into 3 groups; Group 1: 21 young wild type mice varying in age from 10–18 weeks days, group 2: seven Purkinje cell degeneration mice (*pcd*^*5J*^) varying in age from 10–13 weeks days, group 3: six old mice aged 70–71 weeks (*C57BL/6J*) (Table 1). Animals were killed by cervical dislocation in accordance with the humane killing of animals under schedule 1 to the Animals (Scientific Procedures) Act 1986.

**Table 1:** The numbers and age ranges of animals analysed.

Animal type	Number collected	Age range of animals
Wild type	17	10–18 weeks
Aged	6	70–71 weeks
*pcd*^*5J*^	7	10–13 weeks

### Mitochondrial isolation

Whole mouse brain was divided into three sections; cortex, cerebellum and brainstem. Samples were placed in GentleMACS C tubes with mitochondrial extraction buffer (consisting of 50 mM Tris-HCl ph7.4, 100 mM KCl, 1.5 mM MgCl2, 1 mM EGTA, 50 mM HEPES and 100 mM sucrose; all sourced from Sigma) and homogenised using a GentleMACS Dissociator (Miltenyi Biotec). The resulting homogenates were spun at 4°C in an Eppendorf Model 5417R Microcentrifuge (Fisher Scientific); firstly at 850 x g for 10 minutes, then the supernatant obtained was centrifuged separately at 1000 x g for 10 minutes to yield a nuclear pellet and a final spin at 10000 x g for 30 minutes to produce the mitochondrial pellet; the remaining supernatant contained the cytosolic fraction. Additional mitochondrial extraction buffer was added to the mitochondrial pellet and re-spun to obtain a purer fraction of isolated mitochondria. All protein extractions were stored at -80°C.

Mitochondrial extractions from the different brain regions of three wild type mice were prepared fresh, straight after sample collection. The remaining samples were all frozen at -80°C following collection, they were thawed later to extract mitochondrial fractions. This difference in sample preparation was used to help determine whether extracting mitochondria from frozen samples rather than fresh impacts upon the integrity of the mitochondria and the enzyme activity of the sample.

### Protein assay

The protein concentrations of the samples were determined using the Bradford Assay—Sigma [22]. Bovine serum albumin (BSA- Fisher Scientific) standards of known protein concentration varying from 0 to 2 mg/ml were added to Bradford reagent in disposable cuvettes (Fisher Scientific) and the absorbance was measured spectrophotometrically at 595nm in a Thermo Scientific Spectrometer Helios Episilon (Fisher Scientific). The absorbance of the known protein standards were plotted using a linear regression. The mitochondrial fractions were diluted 1 μl in 100 μl Tris Buffer and added to Bradford reagent before the absorbance was measured. The absorbance was plotted against the BSA standard curve to determine the estimated protein concentration (mg/ml).

### Protein determination for the complex 1 assay

The complex 1 spectrophotometric assay used in this study was based upon the protocol by Janssen and colleagues [23], with modifications incorporated from the protocol published by Spinazzi [24]. Janssen did not specify a protein weight to load into the assay, instead recommending a volume of mitochondrial suspension (2.5 μl -> 20 μl), whereas Spinazzi stated a minimum weight of 20 μg -> 50 μg mitochondrial protein. The mitochondrial pellets extracted from some cerebellum and brainstem samples were small, so a protein concentration of 30 μg at the lower end of the suggested range by Spinazzi was selected to ensure the volume of sample required in the assay did not exceed sample availability. In order to measure the whole enzyme activity that could be detected from each sample by this assay, freeze-thaw cycles were incorporated. Two additional freeze-thaw cycles were integrated into the protocol prior to the assay; therefore prior to loading the 30 μg, the sample of mitochondrial extraction had been through five freeze thaw cycles.

### Complex 1 Assay

Mitochondrial fractions underwent five freeze-thaw cycles to disrupt the mitochondrial membranes. The reaction cuvette contained 920 μl from a 100 ml complex 1 buffer stock (containing 25 mmol/L monobasic potassium phosphate, 120 μl of 50 mmol/L dichlorophenolindophenol (DCIP), 100 μl of 1mmol/L Antimycin A, 400 μl of 17.5 mmol/L Decylubiquinone, made up to 100 ml with RO water), 50 μl of 70 g/L fatty acid free BSA and 30 μg of mitochondrial sample (all reagents sourced from Sigma). The volume of mitochondrial extract required to load 30ug was previously determined by Bradford assay as described.

The cuvette volume absorbance was recorded at 600nm and allowed to equilibrate at room temp for 1 minute before the initiation of the reaction by the addition of 20 μl of 10 mmol/L stock of NADH (Sigma) after which the absorbance was recorded every 30s for 2.5 minutes. The complex 1 inhibitor rotenone (Sigma) dissolved in dimethyl sulfoxide at a concentration of 40 mmol/L was added to the reaction volume and mixed vigorously before the absorbance was recorded every 30s for a further 3 minutes.

### Calculating Enzymatic Activity

Enzyme activity was calculated as described by Spinazzi and colleagues [24]. The following equation was used to calculate enzyme activity;

Enzyme activity (nmol/min/mg) = (ΔAbsorbance/min×1,000)/[(extinction coefficient of DCIP×volume of sample used in 1 ml) × (sample protein concentration in mg/ml)]

Extinction coefficient of DCIP: 19.1 mM-1cm-1 (Janssen et al. 2007).

Overall activity reaction rate was determined by calculating enzyme activity following the addition of NADH. The rotenone resistant activity rate was determined by readings following the addition of the inhibitor rotenone. Following rotenone addition the reaction cuvette was allowed to equilibrate for one minute after which changes in absorbance were recorded again. Specific Complex 1 enzyme activity was calculated by subtracting the rotenone resistant activity from the total enzyme activity.

### Data distribution

The nature of the distribution of data for Complex 1 activity within each brain region; cortex, cerebellum and brainstem was determined by use of R (R Core Team (2014). R: A language and environment for statistical computing. R Foundation for Statistical Computing, Vienna, Austria http://www.R-project.org/). The data that were found to be normally distributed were plotted as histograms using parametric methods.

### Statistical analysis

Within each brain region the activity of the various sample types; wild type, *pcd*^*5J*^ and old were presented as a mean average alongside calculated standard deviation and standard error mean. Noted differences amongst the various sample types within each brain region, and between the different regions were assessed for significance. Unpaired t-test’s with Welch’s correction and one-way ANOVA tests were formed on the results using GraphPad Prism version 6 for Windows, GraphPad Software, San Diego California USA, www.graphpad.com.

## Results

### The activity of complex 1 was highest in all the brain regions from wild type mitochondria compared with the aged and *pcd*^*5J*^ brain mitochondria

**Fig 1:**
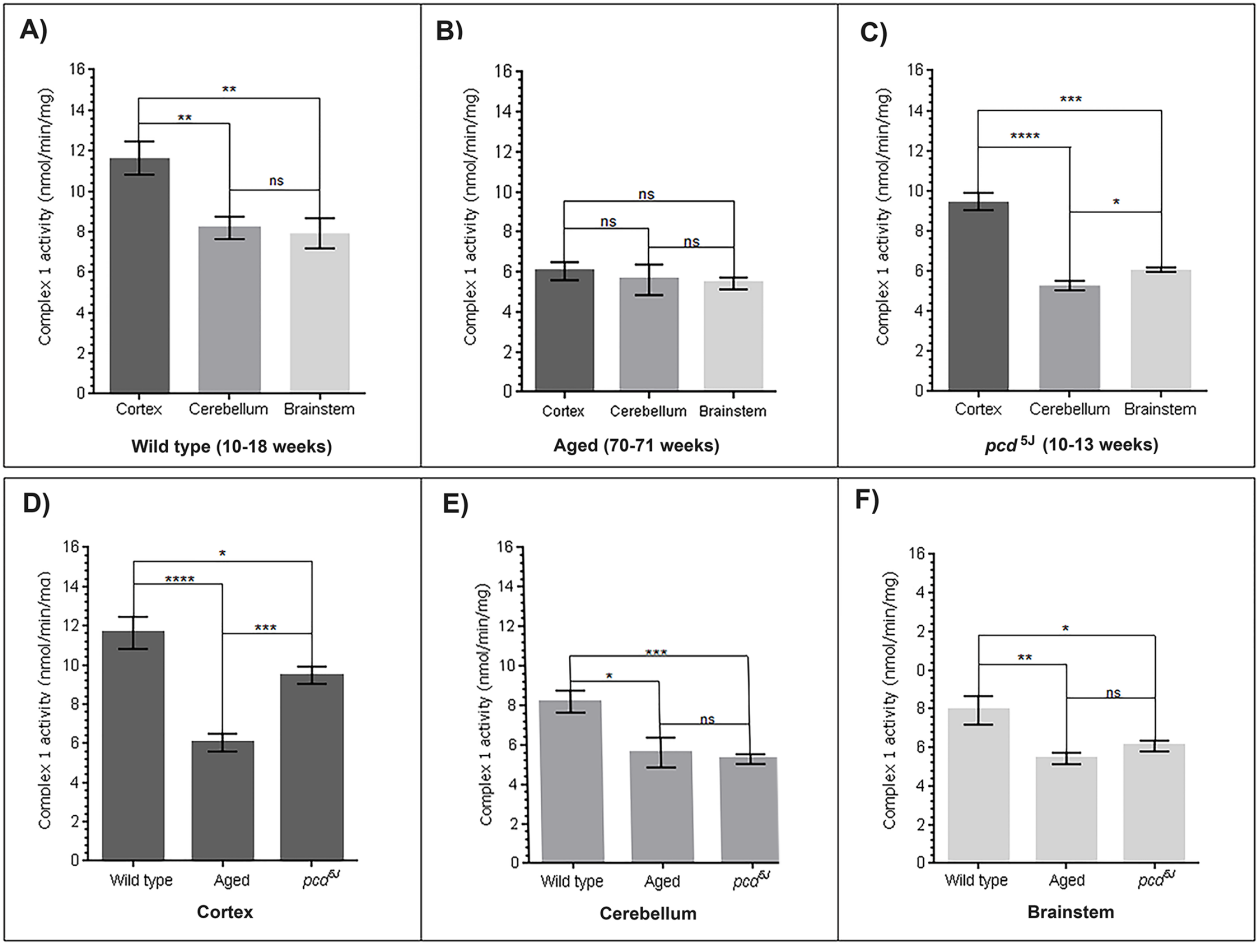
**A) The cortex displays significantly higher complex 1 activity in wild type mitochondria compared to other brain regions**. Complex 1 activity was measured spectrophotometrically in the cortex, cerebellum and brainstem mitochondria of wild type mice aged 10–18 weeks. Complex 1 activity is significantly higher in the cortex compared to the activity in the cerebellum and brainstem of wild type animals. The cerebellum and brainstem mitochondria in wild type animals exhibit similar levels of complex 1 activity. (Cortex n = 11, cerebellum n = 16, brainstem n = 15). **B) In aged animals, complex 1 activity is similar in all three-brain regions**. Complex 1 activity was measured spectrophotometrically in the cortex, cerebellum and brainstem mitochondria of aged mice (70–71 weeks old). The reduction in complex 1 activity in the brain with ageing is not region-specific suggesting that the decrease in activity affects the entire brain. (Cortex n = 6, cerebellum n = 6, brainstem n = 6). **C) Complex 1 activity is significantly reduced in *pcd***^***5J***^ **cerebellum mitochondria**. Complex 1 activity was measured spectrophotometrically in the cortex, cerebellum and brainstem mitochondria from a neurodegeneration mouse model, *pcd*^*5J*^, aged 10–13 weeks. Complex 1 activity in the cerebellum mitochondria is significantly lower than the activity in the cortex and brainstem mitochondria. (Cortex n = 6, cerebellum n = 7, brainstem n = 6). **D) Complex 1 activity is significantly reduced in the aged cortex mitochondria compared with wild type and *pcd***^***5J***^ **cortex mitochondria**. Complex 1 activity was significantly reduced in the *pcd*^*5J*^ cortex compared to the wild type cortex mitochondria. However, the aged cortex mitochondria displayed the lowest complex 1 activity. (Wild type n = 11, aged n = 6, *pcd*^*5J*^ n = 6). **E) Similar levels of complex 1 activity are observed in cerebellar mitochondria from aged and *pcd***^***5J***^ **animals**. Both the aged and *pcd*^*5J*^ mitochondria exhibit reduced levels of complex 1 activity in the cerebellum compared with the wild type cerebellum mitochondria. (Wild type n = 16, aged n = 6, *pcd*^*5J*^ n = 7). **F) Significantly reduced levels of complex 1 activity are observed in the aged and *pcd***^***5J***^ **brainstem mitochondria compared to wild type brainstem mitochondria**. (Wild type n = 15, aged n = 6, *pcd*^*5J*^ n = 6). All assays contained 30mg/ml mitochondrial protein. Replicates were carried out from brain tissue isolated from individual animals. Columns display mean activity ± SEM. * = p<0.05, ** = p<0.03, *** = p <0.02, **** = p <0.01 two-tailed *t*-test with Welch’s correction. ns = no significant difference. Refer to tables 2 and 3 for *p values*.

The wild type brain from mice aged 10–18 weeks were used for the ‘young brain mitochondria’ control group. The *pcd*^*5J*^ mouse represents a model for neurodegeneration. In all brain regions the mitochondria from wild type animals displayed a greater complex 1 activity in comparison to the activity in the aged animals and neurodegeneration model (Fig 1D–1F).

### Complex 1 activity of wild type mice is highest in the cortex mitochondria

Complex 1 activity of the cortex, cerebellum and brainstem of wild type mouse brain produced significant differences (one way ANOVA *p value = 0*.*0015)* (Table 2). Activity was measured to be the greatest in the cortex; significantly higher than cerebellum (*p value = 0*.*0026)* and the brainstem samples *(p value = 0*.*0028)*. No significant difference in Complex 1 activity was measured between wild type cerebellum and brainstem samples (*p value = 0*.*7765)* (Table 2).

**Table 2:** Comparisons of complex 1 between wild-type, aged and *pcd*^*5J*^mouse brain mitochondria.

Animal	Analysis between	Statistical test	*p value*
Wild type (10–18 weeks)	Cortex/Cerebellum	Unpaired t-test with Welch's correction	0.0026*
Wild type (10–18 weeks)	Cortex/Brainstem	Unpaired t-test with Welch's correction	0.0028*
Wild type (10–18 weeks)	Cerebellum/Brainstem	Unpaired t-test with Welch's correction	0.78
Wild type (10–18 weeks)	Cortex/Cerebellum/Brainstem	One-way ANOVA	0.0015*
Aged (70 weeks)	Cortex/Cerebellum	Unpaired t-test with Welch's correction	0.65
Aged (70 weeks)	Cortex/Brainstem	Unpaired t-test with Welch's correction	0.29
Aged (70 weeks)	Cerebellum/Brainstem	Unpaired t-test with Welch's correction	0.82
Aged (70 weeks)	Cortex/Cerebellum/Brainstem	One-way ANOVA	0.72
*pcd*^5J^ (10–13 weeks)	Cortex/Cerebellum	Unpaired t-test with Welch's correction	< 0.0001*
*pcd*^5J^ (10–13 weeks)	Cortex/Brainstem	Unpaired t-test with Welch's correction	0.0003*
*pcd*^5J^ (10–13 weeks)	Cerebellum/Brainstem	Unpaired t-test with Welch's correction	0.017*
*pcd*^5J^ (10–13 weeks)	Cortex/Cerebellum/Brainstem	One-way ANOVA	<0.0001*

### Complex 1 activity of *pcd*^*5J*^ mouse mitochondria is highest in the cortex

Complex 1 activity measured in the *pcd*^*5J*^ mouse brain identified significant differences between the cortex, cerebellum and brainstem mitochondria (one way ANOVA *p value = <0*.*0001)* (Fig 1C and Table 2). Greatest complex 1 activity is in the cortex, and the recorded mean activity is significantly higher than the cerebellum (*p value = <0*.*0001)* and the brainstem samples *(p value = 0*.*0003)*. In contrast with the wild type samples, a significant difference in measured complex 1 activity existed between *pcd*^*5J*^ cerebellum and brainstem samples (*p value = 0*.*017)*. This would be expected for a model of cerebellar degeneration.

### Overall complex 1 activity is similar across all three-brain regions in aged mitochondria

Complex 1 activity measured in the old mice brains produced no significant difference between the cortex, cerebellum and brainstem samples (one way ANOVA *p value = 0*.*72)* (Fig 1B and Table 2). Complex 1 activity is slightly higher in the cortex, but not significantly greater than the recorded activity in the brainstem samples (*p value = 0*.*29)* and that of cerebellum mitochondria *(p value = 0*.*65)*. No significant difference in activity was recorded between the old cerebellum and brainstem samples (*p value = 0*.*82)*.

### Complex 1 activity decreases with age and neurodegeneration

In all three brain regions (cortex, cerebellum and brainstem) the recorded complex 1 activity is significantly decreased in the old mice and *pcd*^*5J*^ when compared with wild type mice. The greatest age related decrease is observed in the cortex (*p value = <0*.*0001))* when comparing wild type to aged mitochondria. Similarly in the *pcd*^*5J*^ mouse mitochondria complex 1 activity is significantly decreased compared to the control, the greatest decrease is observed within the cerebellum (*p value = 0*.*0001)* (Fig 1 and Table 3).

**Table 3:** Complex 1 activity comparison of cortex, cerebellum, and brainstem mitochondria.

Brain region	Analysis between	Statistical test	*p value*
Cortex	Wild type/Aged	Unpaired t-test with Welch's correction	<0.0001*
Cortex	Wild typed/*pcd*^5J^	Unpaired t-test with Welch's correction	0.035*
Cortex	Aged/*pcd*^5J^	Unpaired t-test with Welch's correction	0.0003*
Cortex	Wild type/Aged/*pcd*^5J^	One-way ANOVA	0.0001*
Cerebellum	Wild type/Aged	Unpaired t-test with Welch's correction	0.0197*
Cerebellum	Wild type/*pcd*^5J^	Unpaired t-test with Welch's correction	0.0001*
Cerebellum	Aged/*pcd*^5J^	Unpaired t-test with Welch's correction	0.69
Cerebellum	Wild type/Aged/*pcd*^5J^	One-way ANOVA	0.0025*
Brainstem	Wild type/Aged	Unpaired t-test with Welch's correction	0.0060*
Brainstem	Wild typed/*pcd*^5J^	Unpaired t-test with Welch's correction	0.027*
Brainstem	Aged/*pcd*^5J^	Unpaired t-test with Welch's correction	0.084
Brainstem	Wild type/Aged/*pcd*^5J^	One-way ANOVA	0.054

### Complex 1 activity was measured to be greater in mitochondria extracted from frozen rather than fresh tissues

**Fig 2:**
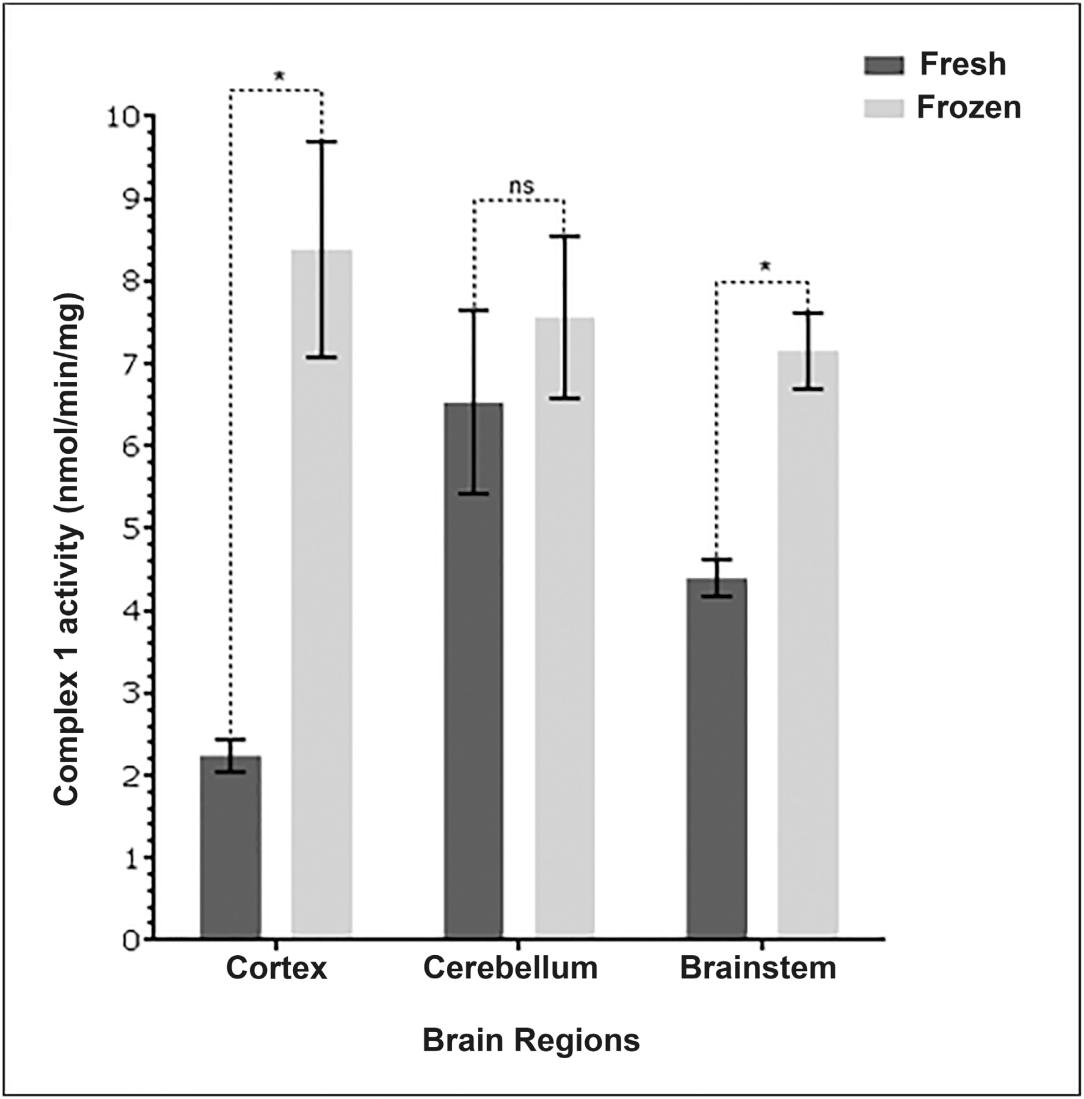
Mitochondria extracted from frozen cortex and brainstem tissue recorded greater complex 1 activity. Mitochondria were extracted from freshly collected and frozen murine brain tissue samples (cortex, cerebellum and brainstem). All assays contained 30mg/ml mitochondrial protein. The activity of complex 1 is found to be significantly greater in mitochondria from frozen cortex and brainstem tissues in comparison to mitochondria extracted from fresh tissue. n = 9 from 3 separate brains (cortex n = 3, cerebellum n = 3, brainstem n = 3). Columns display mean activity ± SEM. * = P <0.05 two-tailed *t*-test with Welch’s correction. ns = no significant difference.

Measured complex 1 activity of wild type cortex and brainstem mitochondria extracted from frozen tissue samples is significantly higher than mitochondria extracted from similar fresh samples (Fig 2). There isn’t a significant difference in complex 1 activity measured in mitochondria extracted from frozen wild type cerebellum compared to fresh (Table 4).

**Table 4:** Comparison of complex 1 activity in mitochondria isolated from either freshly collected tissue or frozen tissue.

Brain region from wild type mouse brain	Unpaired t-test with Welch's correction *p value*
Cortex fresh/frozen	0.0378*
Cerebellum fresh/frozen	0.5272
Brainstem fresh/frozen	0.0137*

## Discussion

Our study aimed to reveal baseline complex 1 activity in the three major compartments of mammalian brain. We looked to see whether there are detectable region specific changes at this level, in response to ageing or neurodegeneration.

We find significant differences in complex 1 activity when comparing distinct regions of the brain. In all sample types; wild type, *pcd*^*5J*^ and old, the cortex was measured to have a higher mean complex 1 activity compared to the cerebellum and brainstem. This contradicts previous work in the rat citing the cerebellum as the area of greatest complex 1 activity [6]. The observed increase in activity in this brain region could be caused by a greater amount of complex 1 present in the mitochondria in the cortex. Complex 1 is composed of many subunits, and some genes encoding for these subunits, for example the Ndufa10 gene are seen to have a higher expression in the cortex when compared to the cerebellum and pons (S1 Fig).

We found complex 1 activity to be significantly diminished in cortex, cerebellum and brainstem mitochondria isolated from aged mice when compared to the wild type mice used as a young control group. A number of studies have previously reported similar findings [6], [7], [25], [26], [27]. This is contradictory to previous research that has shown no significant decline in complex 1 activity in brain tissues [28]. The significant decline in measured complex 1 enzymatic activity in the brain could be caused by a reduction in mitochondrial functionality with age; supporting the hypothesis of the ‘mitochondrial theory of ageing’ [29].

The greatest decrease in complex 1 activity with age was measured in the cortex when comparing wild type and old brain samples. This finding is contradicted by previous work that noted a 30% decline in the complex 1 activity of the cortex; much lower than the 50–60% in the brainstem [6]. Our findings suggest that complex 1 in the cortex undergoes a greater decrease in functionality in ageing brain. Many of the 45 subunits making up complex 1, are encoded for by mitochondrial DNA, which may be more susceptible to oxidative damage. One of the mitochondrial DNA subunits of complex 1 is encoded for by the mitochondrial encoded NADH:ubiquinone oxidoreductase core subunit 3 gene (MT-ND3). In situ hybridisation (ISH) of mouse cortex has been shown to display a higher density of MT-ND3 expression in contrast to the cerebellum; potentially supporting the hypothesis of a greater target mass of complex 1 in the cortex in comparison to other brain regions (supplementary data)[30].

The cortex has substantially decreased functionality with age compared with the cerebellum and brainstem. The hind brain regions appear to age similarly; exhibiting no significant difference between the brain regions of the wild type control and the old samples. However the similar pattern of complex 1 activity change seen in ageing of the cerebellum and brainstem is not reflected in the neurodegeneration model. The complex 1 activity measured in the *pcd*^*5J*^ cerebellum is significantly lower than the brainstem, suggesting that complex 1 function is affected to a greater extent in the cerebellum which is where the neurodegeneration occurs in this model. The cerebellum has been shown to contain significantly lower mitochondrial DNA copy numbers in comparison to other brain regions (Fuke et al 2011). Mutations to mitochondrial DNA may have a greater effect in regions of the brain that are low in mitochondrial DNA copy number. The reduced function of complex 1 in the cerebellum is unsurprising considering the neurodegeneration model used. The *pcd*^*5J*^ phenotype is characterised by a dramatic and rapid loss of Purkinje cells in the cerebellum [16]. Purkinje cells contain mitochondria, so destruction of the cells will be correlated to a loss of mitochondria from the cerebellum. A loss of mitochondria equates to a loss of complex 1. It could be suggested that this loss of mitochondria from the cerebellum is sufficient enough to cause a significant decrease of complex 1 activity. However, this would infer that the majority of complex 1 activity in the cerebellum is housed in the Purkinje cells which make up a small percent of the overall cerebellar volume.

An unexpected result of this study was that complex 1 activity was measured to be greater in mitochondria extracted from samples which were frozen upon collection, rather than in the mitochondria extracted from fresh samples. Previous studies have recommended the use of fresh mitochondrial extractions in order to maximise enzyme activity [31], [32]. A possible explanation is that the additional initial freeze-thaw cycle the ‘frozen samples’ underwent had the beneficial effect of releasing an increased number of mitochondria from within the cell membrane [33].

## Conclusions

The cortex was determined as the brain region exhibiting the greatest mitochondrial complex 1 activity. We show that complex 1 activity decreased with age, which supports the mitochondrial theory of ageing and promotes further research into a mitochondrial, in particular complex 1, role in the aetiology of ageing.

The *pcd*^*5J*^ mouse exhibited a decrease in complex 1 activity compared to the control group. Our findings support previously published research exhibiting a diminished complex 1 activity in the *pcd*^*5J*^ mouse model [21]. The significant decrease of activity in the cerebellum demonstrates that the loss of Purkinje cells has a negative impact upon the mitochondrial function of the cerebellum as a whole. Throughout this study we have measured and compared equal quantities of mitochondrial protein from each region of the brain. Therefore our conclusion must be that the activity we measure is not per brain region but per mitochondrial unit. This needs to be taken into consideration when analysing brain tissues for mitochondrial content, in that increased numbers of mitochondria may not correspond proportionately to increased activity and ATP production.

### Ethical approval

Animals were bred and housed in accordance with strict Home Office stipulated conditions. The overall programme of work (in respect to the original UK Home Office Project Licence application) is reviewed by the Animal Welfare and Ethical Review Body at the University of Nottingham and then scrutinised by the UK Home Office Inspectorate before approval by the Secretary of State. Individual study protocols link to the overarching Home Office Project Licence and are made available to the Named Animal Care and Welfare Officer, the Named Veterinary Surgeon (both are members of the AWERB), the animal care staff and the research group. The Project Licence Number for the breeding and maintenance of this genetically altered line of mice is PPL 40/3576. The mice are typically group housed and maintained within solid floor cages containing bedding and nesting material with additional environmental enrichment including chew blocks and hiding tubes. Cages are Individually Ventilated Cage Units within a barrier SPF unit to maintain bio-security. Animals are checked daily by a competent and trained animal technician. Any animal giving cause for concern such as subdued behaviour, staring coat, loss of weight or loss of condition will be humanely killed using a Home Office approved Schedule 1 method of killing.

## Supporting Information

S1 Fig **A) The gene expression of NDUFA10 (NADH:ubiquinone oxidoreductase subunit a10) is higher in the cortex than the cerebellum and pons**. NDUFA10 gene expression in mouse (images and data from Allen Mouse Brain Atlas). Raw expression value of NDUFA10 of 13.78 in the cortex (I), 8.37 in the cerebellum (II) and 9.00 in the pons (III). **B) The gene expression of NADH oxidoreductase core subunit 3 (MT-ND3) is higher in the cortex than in the cerebellum**. MT-ND3 gene expression in mouse in situ hybridisation from the Allen Mouse Brain Atlas. Raw expression value in cortex (I) 14.91, lower expression of 10.67 in cerebellum [30]. Website: 2015 Allen Institute for Brain Science. Allen Mouse Brain Atlas [Internet]. Available: http://mouse.brain-map.org. NDUFA10: http://mouse.brain-map.org/gene/show/43116 and MT-ND3: http://mouse.brain-map.org/gene/show/17485.
(TIF)
