# Supplementary figures and images for: Mitochondrial Complex 1 Activity Measured by Spectrophotometry Is Reduced across All Brain Regions in Ageing and More Specifically in Neurodegeneration
Source: PLoS One. 2016 Jun 22;11(6):e0157405. doi: 10.1371/journal.pone.0157405 (PMC4917223; doi:10.1371/journal.pone.0157405)

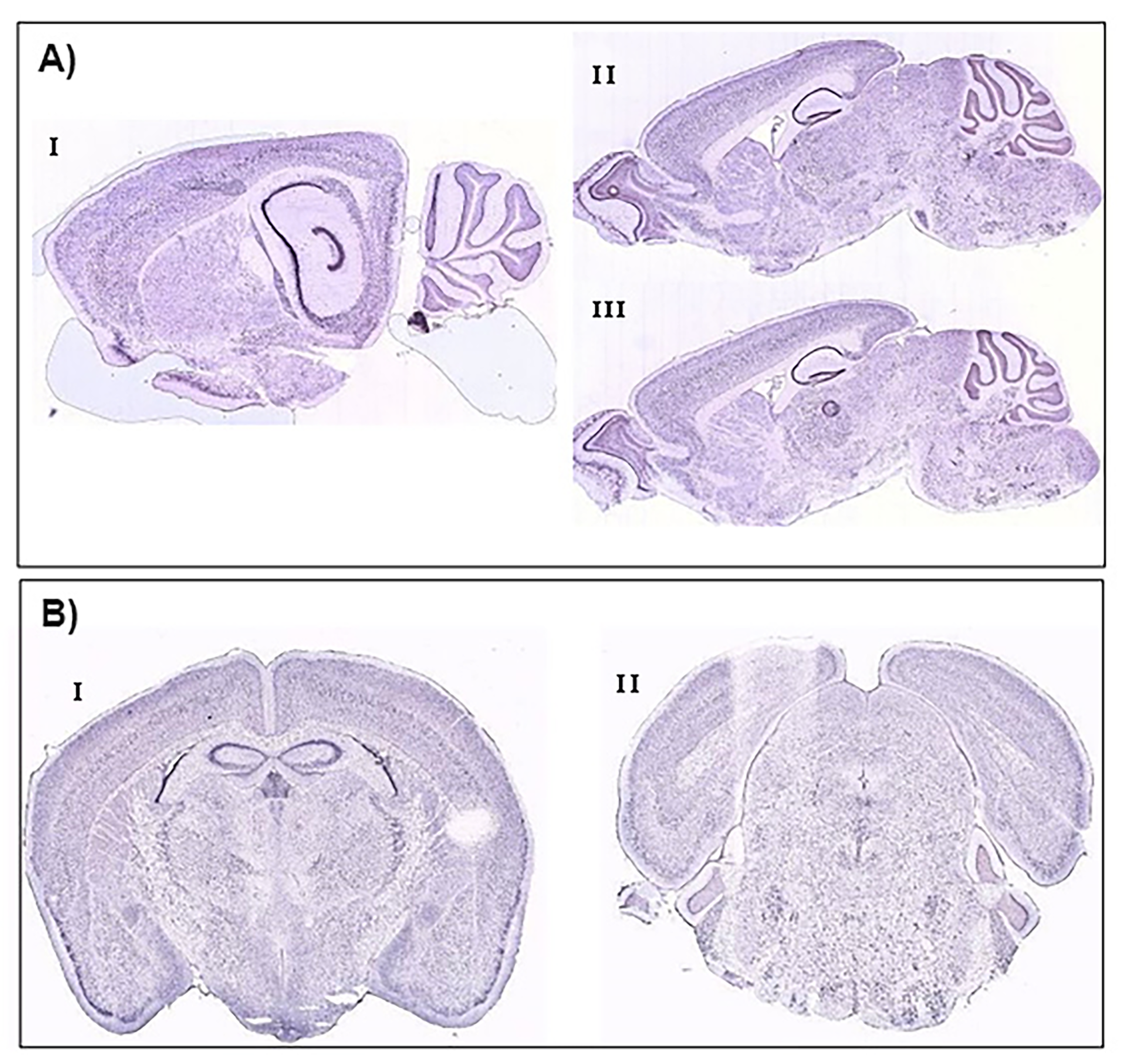

Supplement: S1 Fig — A) The gene expression of NDUFA10 (NADH:ubiquinone oxidoreductase subunit a10) is higher in the cortex than the cerebellum and pons. NDUFA10 gene expression in mouse (images and data from Allen Mouse Brain Atlas). Raw expression value of NDUFA10 of 13.78 in the cortex (I), 8.37 in the cerebellum (II) and 9.00 in the pons (III). B) The gene expression of NADH oxidoreductase core subunit 3 (MT-ND3) is higher in the cortex than in the cerebellum. MT-ND3 gene expression in mouse in situ hybridisation from the Allen Mouse Brain Atlas. Raw expression value in cortex (I) 14.91, lower expression of 10.67 in cerebellum [30]. Website: 2015 Allen Institute for Brain Science. Allen Mouse Brain Atlas [Internet]. Available: http://mouse.brain-map.org. NDUFA10: http://mouse.brain-map.org/gene/show/43116 and MT-ND3: http://mouse.brain-map.org/gene/show/17485. (TIF) [file pone.0157405.s001.tif]
